# Association of globalization with the burden of opioid use disorders 2019. A country-level analysis using targeted maximum likelihood estimation

**DOI:** 10.1186/s12992-023-00980-3

**Published:** 2023-10-16

**Authors:** Guillaume Barbalat, Geeta Reddy, Nicolas Franck

**Affiliations:** 1 Centre ressource de réhabilitation psychosociale et de remédiation cognitive, Pôle Centre rive gauche UMR 5229, Hôpital Le Vinatier, CNRS & Université Lyon 1, Lyon, France; 2 International Committee of the Red Cross (ICRC), 19, Avenue de la Paix, Geneva, 1202 Switzerland

**Keywords:** Opioid use disorders, Opioid crisis, Opioid epidemics, Globalization, Global burden of Disease, Socio-economic factors

## Abstract

**Background:**

The “opioid crisis” has been responsible for hundreds of thousands deaths in the US, and is at risk of dissemination worldwide. Within-country studies have demonstrated that the rise of opioid use disorders (OUD) is linked to increased access to opioid prescriptions and to so-called “diseases of despair”. Both have been related to the emergence of globalization policies since the 1980s. First, globalized countries have seen a reorganization of healthcare practices towards quick and easy answers to complex needs, including increased opioid prescriptions. Second, despair has gained those suffering from the mutations of socio-economic systems and working conditions that have accompanied globalization policies (e.g. delocalization, deindustrialization, and the decline of social services). Here, using data with high quality ratings from the Global Burden of Disease database, we evaluated the country-based association between four levels of globalization and the burden of OUD 2019.

**Results:**

The sample included 87 countries. Taking into account potential country-level confounders, we found that countries with the highest level of globalization were associated with a 31% increase in the burden of OUD 2019 compared to those with the lowest level of globalization (mean log difference: 0.31; 95%CI, 0.04–0.57; p = 0.02). Additional analyses showed a significant effect for low back pain (mean log difference: 0.07; 95%CI, 0.02–0.12; p = 0.007). In contrast, despite sharing some of the risk factors of OUD, other mental and substance use disorders did not show any significant relationship with globalization. Finally, socio-cultural de jure globalization, which compiles indicators related to gender equality, human capital and civil rights, was specifically associated with the burden of OUD (mean log difference: 0.49; 95%CI: 0.23,0.75; p < 0.001).

**Conclusions:**

These findings suggest that OUD may have inherent underpinnings linked to globalization, and more particularly socio-cultural aspects of globalization. Key factors may be increased rights to access prescriptions, as well as increased feelings of despair related to the erosion of local cultures and widening educational gaps.

**Supplementary Information:**

The online version contains supplementary material available at 10.1186/s12992-023-00980-3.

## Introduction

Opioid analgesics play a key role in the treatment of acute pain and palliative care. Yet, when prescribed inadequately, opioids may be associated with negative outcomes, such as dependence or overdose death. In the US, the so-called “opioid epidemics” refers to the fact that opioid misuse and overdoses are at epidemic levels, with nearly 500,000 overdose deaths between 1999 and 2019 [1]. The opioid crisis however, is not solely a US problem. According to the Global Burden of Disease (GBD) estimates, high-income countries or territories have seen an impressive rise of more than 250% in the burden of opioid use disorders (OUD) since the 1990s [2]. Low- and middle-income countries are showing great geospatial disparities, with increases above 30% in countries from North Africa, the Middle East, or Latin America; more modest increases in South and Central Asia, Central and Eastern Europe; and decreases in countries from Southeast and East Asia, or Sub-Saharan Africa [2]. More importantly, some have argued that there is a real potential for the opioid epidemic to spread to lower- and middle-income countries [3–5]. Given that these countries possess very few resources for effective treatment and prevention, this could have dramatic consequences on people and healthcare systems.

To avoid further dissemination of the crisis, it is crucial to better understand its determinants. As a mental and behavioral disorder, OUD has been found to be associated with typical upstream socio-economic determinants of mental health such as unemployment, socio-economic inequalities, or urbanization [6, 7]. Within-country studies, mostly carried out in the US, have suggested three other prominent features involved in the rise of OUD. First, opioids have been largely and incautiously over-prescribed in profit-making practices that typically over-rely on quick and easy answers to complex physical and psychological needs [8, 9]. Increased rates of opioid prescriptions are also thought to be the result of unrealistic patient demands regarding pain elimination in the wider context of a consumerist culture [8]. Second, pharmaceutical conglomerates have used aggressive marketing strategies to facilitate the dissemination of opioid prescription [10, 11], and have been subtly involved in the shaping of education regarding opioid prescriptions [8]. Third, heavy opioid consumption has been linked to so-called “diseases of despair”, expressed as a result of economic and socio-structural changes of modern societies [12–16]. These changes pertain to lack of economic opportunity for middle-class workers, due to deindustrialization and de-localization, associated with declining welfare and social services. Another underpinning of diseases of despair is lack of educational attainment, which in meritocratic societies is associated with lower socio-economic status and feelings of humiliation.

Overall, some of the major contributors of the burden of OUD may be associated with the structural changes that have accompanied the emergence of neoliberal ideologies since the 1980s [17]. Economically, these changes pertain to unrestrained search for profit, the promotion of free market competition, and other socio-economic choices involving liberalization, privatization, deregulation, delocalization, deindustrialization, and declining state services [17]. Culturally, these changes are thought to be related to individualism [17] and consumerism [8], but also widening educational gaps in meritocratic countries, where social esteem is based on human capital [18].

Unfortunately, the conjunction of these socio-economic and structural aspects are not specific to the US. For instance, Palinkas discusses the opioid epidemic spreading from the US to Mexico as a “global problem”, linked to “cultural changes that predispose individuals to abuse of prescription medication and other opioids” [4]. The article mentions that one important aspect of such cultural changes is “exposure to US society”, and in particular “acculturation of values, attitudes, and behaviors” [that increases] “the need and the desire to use opioids” [4]. Likewise, Humphreys points to the danger of the “globalization of the prescription opioid addiction and overdose epidemic”, due to pharmaceutical companies expanding sales of prescription opioids, especially in countries with loose regulations [5]. Finally, Friedman et al. expressively point to the risk of spread of the opioid crisis to non-US globalized countries that are also impacted by neoliberalization, a risk that is mitigated by better prescribing regulations or unionization [17].

Recently, the propagation of neoliberal policies has been linked to a rapid increase of economic globalization since the 1980s [19], where increasing trade and financial flows have been accompanied by liberalization, privatization, disengagement from the State, and a free-market, deregulated economy. As explained above, these may precipitate what we referred to as diseases of despair, and thus OUD. These may also promote profit-making clinical practices to over-prescribe opioids, pharmaceutical companies to develop aggressive marketing strategies, and patients to over-demand and consume opioids. It is noteworthy however, that while related, both concepts of globalization and neoliberalism are not strictly similar. For instance, the US is seen as a neoliberal economy, yet, has been consistently scoring in the mid-high (and not high) range in globalization measures. This is because, as others have argued, “as a large economy, a high proportion of its trade is internal which means that the USA does not “need” to be as globalized as small countries” [20].

Importantly, if globalization is tied to neoliberal policies, then one may hypothesize that globalization is also a core structural determinant of OUD. Globalization is not restricted to economic activities however, but refers to a whole “process of interaction and integration among people, companies, and governments worldwide” [21]. Other than its economic aspects, globalization encompasses socio-cultural and political facets, measured by the spread of technology, information, but also people, ideas, political influences, and cultures, across countries. Interestingly, these aspects of globalization may be linked to diseases of despair, e.g. via increased automation [22], destruction of local cultures [23, 24], or educational divides [25].

Despite the use of the terms “global” or “globalization” in the reviews, opinions and personal views mentioned above, research on social determinants of OUD has never empirically tested whether socio-economic and cultural facets of globalization had any impacts on OUD worldwide. Yet, whether globalization correlates with OUD is an important question: if globalization indicators were to be associated with OUD, then globalizing countries should expect a rise in OUD and should therefore prepare their healthcare systems accordingly, develop mitigation strategies, or even perhaps question globalization itself.

The current observational, population-level study, aimed to investigate the relationship between globalization and OUD. In addition, because some of the determinants of OUD that may be linked to globalization (e.g. despair) overlap with those of low back pain and other mental and substance use disorders, we wished to determine whether the latter disorders were also related to globalization. Finally, because globalization is a broad concept that encompasses various indicators, we explored the relationship between globalization and OUD across its economic, socio-cultural and political facets.

Collecting data on opioid burden is particularly prone to measurement bias (e.g. due to reporting bias). Measurement error in OUD might also correlate, positively or negatively, with other socio-economic or demographic variables. Another caveat is that the temporal relationship between globalization and OUD is probably not perfectly linear. In fact, the relationship between globalization and OUD may be very complex, in that it would depend on delayed and cumulated effects, as well as interactions and feedback loops with other variables, such as unemployment or inequalities. Finally, in observational studies the careful choice of an appropriate set of covariates to include in a statistical model is of paramount importance. On the one hand, one needs to consider a set of potential confounders that may affect both the exposure (globalization) and outcome (OUD). Not adjusting for these variables would bias the estimation of the effect of globalization on OUD, as the latter would include the effect of the omitted variables – the rationale for our choice of country-level confounders is reported in the Methods below. On the other hand, since our study aimed to investigate the total effect of globalization on OUD, potential mediators of this relationship, i.e. mechanisms by which globalization is linked to OUD, should not be included in the statistical model. As mentioned above, these may include aspects of neoliberal globalization that were related to OUD, including a free-market, deregulated economy favoring delocalisation, deindustrialization, the rise of profit-making clinical practices and pharmaceutical conglomerates, as well as over-consumption from citizens.

First, to investigate whether globalization is associated with OUD, we used high-quality country-level estimates from the GBD study 2019 [26], which present the notable advantage of providing measures of opioid burden that rely on multiple temporal and geographical data sources, as well as robust modeling techniques. We reasoned that this strategy should reduce (though certainly not take out) the risk of measurement bias. Second, because globalization is related to other macro-socio-economic and demographic indicators typically involved in mental and substance use disorders (e.g. unemployment, inequalities), we aimed to obtain estimates of the effect of globalization while controlling for these background characteristics. Third, in theory, modeling temporal dependencies between our exposure, outcome and covariates would have increased the precision of our estimators, yet it would also have seriously complexified our analysis. Therefore, we chose to not model temporal dependencies and instead consider cross-sectional relationships between globalization, covariates, and the burden of OUD, using a one-year lag (2018 vs. 2019). Fourth, our analytical strategy took advantage of targeted maximum likelihood estimation and machine learning algorithms [27], which address some of the statistical challenges inherent to the estimation of the association between an exposure and outcome in observational studies.

## Methods

### Data

#### Presentation of the global burden of disease database

We extracted our outcome variables and some of our covariates from the GBD database [26]. This data source has been described as the most comprehensive health database worldwide. It provides estimates of 369 diseases and injuries for 204 countries and territories, using more than 86,000 data sources from 1990 to 2019. The GBD also provides a number of covariates measuring socioeconomic, demographic, health system access, climate, and food consumption indicators.

Primary data sources include a comprehensive catalogue of health-related data such as surveys, studies from the scientific literature, censuses, registries, and other administrative data. To be included in the GBD study, input sources have to comply with guidelines provided by GATHER which are the gold standard for observational studies [28]. Following GATHER ensures that adequate information is available to assess the quality of the source data, in particular that study samples are representative of the general population and have clinical thresholds established by international classifications.

All available data are standardized, mapped to the GBD cause list, stratified by age and sex, corrected for miscoded causes and redistributed to appropriate causes, aggregated over various sources, and finally pooled into a single database [26].

The number of sources available in the GBD database to provide estimates for each condition was as follows: 2569 for OUD; 455 for low back pain; 3944 for alcohol use disorders; 197 for anxiety disorders; 147 for bipolar disorder; 461 for cannabis use disorders; 528 for depressive disorders; 1949 for eating disorders; and 202 for schizophrenia [29]. Note that OUD, alcohol use disorders, and eating disorders have sources for fatal in addition to non-fatal outcomes, which the other disorders have not.

These relatively large numbers are mitigated by two caveats. First, available data may be of poor quality, for instance if it does not use the preferred case definition or an appropriate measurement method. Using a 5 stars classification system, the GBD provides a grading of data quality for each country, which depends on data availability, completeness, detail of mortality data and percentage of deaths coded to ill-defined codes or highly aggregated causes [26]. In the current study, we only included data from locations whose data quality was rated with at least 3 stars (N = 87 countries from the original 204 locations where DALYs were estimated; Table 1).

Second, the availability of the data sources is unequally distributed geographically and temporally. To generate robust cause-specific estimates by age, sex, year, and location, the GBD uses powerful statistical modelling techniques that incorporate external data (e.g. from other locations and over time), and enforce the consistency between epidemiological parameters. A detailled description of these methods is provided elsewhere [26, 30–32].

#### Outcome

Our outcome variable was the burden of OUD 2019. Subsequent analyses involved the burden of low back pain and that of other adult mental and substance use disorders for the same year. These were extracted from estimates of the GBD study 2019 as the 2019 age-standardized Disability-Adjusted Life Years rates per 100,000 inhabitants (hereby 2019 DALYs) [26]. Mental and substance use disorders included in the study were: alcohol use disorders, anxiety disorders, bipolar disorder, cannabis use disorders, depressive disorders, eating disorders, schizophrenia.

Briefly, for a specific year, country and disability, DALYs are a measure of overall disease burden, expressed as the sum of the number of estimated Years Lived with Disability (YLD) and early death (Years of Life Lost, YLL). YLD is measured according to the formula YLD = Prevalence x Disability Weights. Disability weights are based on population surveys to lay descriptions of sequelae highlighting major functional consequences and symptoms [26]. Disability weights are held invariant between age and sex groups, as well as locations and over time. Disability Weights are measured on a scale from 0 (full health) to 1 (death). Importantly, in the GBD study, each disability is collectively exhaustive and exclusive of any comorbidity.

Outcomes were log-transformed to allow interpretation of coefficients as percentage differences, and to account for possible non-gaussian distributions.

#### Exposure

The Globalization Index was extracted from the KOF Swiss Economic Institute [33]. The KOF is a “Konjunkturforschungsstelle” which in German means “Business Cycle Research Center”. The Globalization Index measures *de facto* and *de jure* economic, social and political dimensions of globalization for the period 1970 to 2019 on a scale of 1 to 100. As of 2019, it is available for 195 countries. To calculate the Index value, different variables are used and are aggregated using statistically determined weights (obtained from principal component analysis) [33].

The sub-​segment of economic globalization comprises:

- Trade flows, which itself comprises:
  - *de facto* trade globalization, determined on the basis of trade in goods and services, as well as trade partner diversification;
  - *de jure* trade globalization, which includes tariffs, taxes and trade restrictions.

- Financial flow:

- *de facto* financial globalization: foreign direct investment, portfolio investment, international debt, international reserves, international income payments. International debt in particular is defined as the sum of inward and outward stocks of international portfolio debt securities and international bank loans and deposits (as a percentage of the GDP);

- *de jure* financial globalization: investment restrictions, capital account openness, and international investment agreements.

The sub-​segment of social globalization comprises:

- Interpersonal contacts:
  - *de facto*: international telephone connections, transfers, tourism flows and migration;
  - *de jure*: telephone subscriptions, international airports and visa restrictions.

- Information flows:
  - *de facto*: international patent applications, international students, and high-​technology trade;
  - *de jure*: access to television and the internet, press freedom, and international internet connections.

- Cultural globalization:
  - *de facto*: trade in cultural goods, registrations of international trademark rights, trade in personal services, and the numbers of McDonald’s restaurants and IKEA stores;
  - *de jure*: civil rights, gender equality and expenditure on education.

The sub-segment political globalization comprises:

- *de facto*: number of embassies, international non-governmental organizations (NGOs) and participation in UN peacekeeping missions;
- *de jure*: membership of international organizations and international treaties, number of partners in investment treaties.

The KOF provides a comprehensive file that describes the sources and definitions of each sub-segment of globalization (https://ethz.ch/content/dam/ethz/special-interest/dual/kof-dam/documents/Globalization/2018/Globalisation%20Index%202018_1.zip).

We used the KOF Globalization Index (KOFGI) for the year 2018, and further discretized values into 4 different levels (Table 1):

- The lowest level of globalization included 23 countries with a globalization index between 41 and 64 (Low; [41, 64]; N = 23 countries);
- The middle-low level of globalization included 23 countries with a globalization index between 64 (excluded value) and 72 (Mid-Low; (64,72]; N = 23 countries);
- The middle-high level of globalization included 23 countries with a globalization index between 72 (excluded) and 82.5 (Mid-High; (72,82.5]; N = 19 countries);
- The highest level of globalization included 23 countries with a globalization index between 82.5 (excluded) and 91 (High; (82.5,91]; N = 22 countries).

Further analysis investigated the above-mentioned sub-indices of globalization, which we also discretized into 4 different levels of increasing magnitude.

#### Covariates

##### Confounders

Confounders impact both the exposure (economic, socio-cultural and political aspects of globalization) and outcome (the burden of OUD), and are crucial to include in a statistical model to avoid biases in parameter estimates.

We included the following country-level confounders:

- A country’s level of development. Broadly speaking, development is associated with a high standard of living, a high level of industrialization, and advanced technological infrastructure [34]. Specifically, developed economies are associated with efficient transportation infrastructure, telecommunication and information systems, good labor skills, and political stability, that in turn are thought to attract foreign investors [35, 36], and would also favor economic and socio-cultural globalization.

On the other hand, a country’s level of development has been associated with the rate of opioid analgesic consumption [37, 38]. Likewise, as mentioned above, high-income countries have seen a dramatic increase in the burden of OUD compared to low- and middle-income countries [2], suggesting that socioeconomic status and development may be related to OUD [39].

We extracted the Socio-Demographic Index (SDI) for the year 2018 from the GBD database [40] as an index of the level of development of a country. The SDI is a composite indicator of development status, and is measured as the geometric mean of indices of total fertility rate, mean education for those aged 15 and older, and lag distributed income per capita. Its range is 0–1, with values closer to 1 indicating a higher level of development.

- Unemployment rate. A low rate of unemployment would increase the spending power of individuals and be an indicator of a country’s growing economy as well as social stability. In contrast, a high rate of unemployment would signal a failure to utilize the available labor force, i.e. a stagnating, non-resilient economy. According to these accounts, foreign investors would favor countries with a low unemployment rate – provided that they have highly skilled and available employees [41, 42].

On the other hand, unemployment rates have been related to increased opioid prescribing and misuse, and higher overdose mortality [43, 44].

We extracted the Unemployment rate for the year 2018 (unemployed citizens as a percentage of the labor force) from the World Bank database [45].

- Income inequality. Income inequality might drive decreased globalization. Indeed, in countries with a high level of inequality, the poor and middle classes incur reduced spending power and may take on debt for consumption, increasing economic, social and political instability. Inequality also causes increased rents and decreased productive activity, reducing growth and development [46]. Overall, these might lead to reduced foreign investments, as well as lower rates of social and political aspects of globalization.

On the other hand, income inequalities have been related to the burden of OUD. For instance, in Canada, people living in lower-income areas have been found to experience higher rates of opioid-related harms [47]; and in the US, areas with greater income inequality have higher rates of overdose deaths [48].

We extracted a measure of income inequality for the year 2018, as measured by the p90p100 index, from the World Inequality Database [49]. This measure represents the ratio of individuals whose income belongs to the top 10% of the population, divided by the entire population. Its range is 0–1, with values closer to 1 indicating a higher level of inequality.

- Urbanization. Cities are described as the “theaters where globalization plays out” [50], where people, capital, information, services and goods converge to enable economic and socio-cultural globalization [50].

On the other hand, urbanization has been linked to OUD. In the US, while all states saw an increase in opioid-related harms [51], some have reported that a larger proportion of people suffering from OUD may originate from rural (vs. metropolitan areas) [51, 52].

We extracted an index of urbanization for the year 2018 from the GBD database (from 0, low degree of urbanity to 1, high degree of urbanity) [53].

- A pre-exposure measure of the outcome. Here, a pre-exposure measure of OUD cannot be considered, *stricto sensu*, as a confounder as it may not directly affect globalization. However, it is often (if not always) advised to include a pre-exposure outcome value in a statistical model [54–56], as it may be related to an underlying source that might also affect the exposure. Examples of such underlying sources may be historical, geographical or cultural factors both related to globalization and the burden of OUD, e.g. typically factors linked to liberalism and consumerism.

We extracted a pre-exposure measure of the outcome (log-transformed 1990 age-standardized DALYs for OUD – but also all other outcomes tested in this study) from the GBD database [26].

- Other important determinants of globalization, especially economic globalization, may be: quality of institutions and economic structures, market size, trade openness, tendency to tax economic actors, or labor cost [35, 57, 58]. However, we reasoned that these were not obviously related to the burden of OUD (other than via other confounders mentioned above and already included in our statistical model).

##### Factors aiming to improve the precision of the parameter estimates

Factors aiming to improve the precision of the parameter estimates are those factors that are only related to the outcome. We hypothesized such factors to be:

- Children sexual abuse [59]. From the GBD database, we extracted the age-standardized summary of exposure value (SEV) for children sexual abuse for the year 2018, which measures a population exposure to children sexual abuse and takes into account the contribution of that risk to disease burden [53]. SEV is reported on a scale from 0 to 100%; SEV takes the value zero when there is no excess risk for a population and the value 100 when the population is at the highest level of risk.

- Quality of measurement of the outcome. We used the data quality rating system from the GBD database [25].

- Access to and quality of healthcare services [52]. We extracted an index of access to and quality of healthcare services for the year 2018 from the GBD database [53]. Such an index ranges from 0 (low quality of healthcare services) to 100 (high quality).

##### Mediators

Key mediators of the relationship between globalization and OUD were discussed above as being linked to liberalization, privatization, disengagement from the State and, overall, a free-market, deregulated economy. In turn, these are thought to promote: (1) profit-making clinical practices that over-prescribe opioids; (2) pharmaceutical companies that aggressively marketize opioid products; (3) over-demand and over-consumption of opioids from patients; (4) companies that de-localize industrial activities to countries with cheaper workforce; and (5) disengagement from unions, and more generally public regulations and policies services, that fail to protect citizens from financial and economic losses, but also from occupational issues (including occupational pain and diseases). It is important to note that as these factors may be on the causal path between globalization and OUD, they should not be included as covariates in our statistical model. Indeed, adjusting for these factors would under-estimate the total effect of globalization on OUD.

### Analysis

#### Methodological strategy

Our methodological strategy consisted in the following steps:

1. For each observation$i$, we predicted log 2019 DALYs $\widehat{DALY{s}_{i}}$ at each of the four levels of globalization, taking into account our set of covariates. Estimation relied on targeted maximum-likelihood estimation (TMLE) and machine learning (ML) (see estimation strategy below) [27]. Briefly, to reach $\widehat{DALY{s}_{i}}$, observed (log-transformed) outcome values $DALY{s}_{i }$were initially estimated based on our set of covariates. Initial predictions were then updated using inverse probabilities of exposure (a.k.a. weights) to the four levels of globalization (which were themselves predicted based on the covariates).
2. To ensure that our analyses did not suffer from positivity violations, we verified that each country had some probability (i.e. a positive probability) of being exposed to various levels of globalization given their characteristics [60–62]. Probabilities of exposure were predicted using ML algorithms, scaled by the marginal probability of exposure (a.k.a. stabilized) [62], and were trimmed to the 99.9th percentile.
3. Taking the lowest level of globalization (Low) as the reference, we estimated how increasing levels of globalization were associated with our (updated) predictions of 2019 DALYs (mean log difference: High, Mid-High, Mid-Low vs. Low) using the following linear equation:

$$\widehat{DALY{s}_{i}}\equiv {\beta }_{0}+{\beta }_{1}Glo{b}_{1i}+{\beta }_{2}Glo{b}_{2i}+{\beta }_{3}Glo{b}_{3i}$$

, where:

- $Glo{b}_{1i},Glo{b}_{2i}, Glo{b}_{3i}$are dummy regressors which, for each observation $i$, take either values $1$ or $0$ according to whether the observation belongs to the High, Mid-High or Mid-Low globalization level, respectively;
- ${{\beta}}_{1}, {\beta }_{2}, {\beta }_{3 }$are the adjusted marginal effect, in percentage, of increasing levels of Globalization (from Low to High, Mid-High and Mid-Low, respectively), on predictions of 2019 DALYs.

#### Estimation strategy

##### Targeted maximum likelihood estimation

TMLE is a doubly-robust estimator, which relies on the estimation of (1) the outcome regression (where outcome values are predicted based on our set of covariates); and (2) the exposure mechanism (where probabilities of exposure are predicted based on the covariates). Both steps are further integrated by updating (“targeting”) the initial estimation of the predicted outcome based on each country’s inverse probability of exposure (a.k.a. weights) [27]. This aims to incorporate information from the exposure mechanism and create a pseudo-random population of observations with respect to the distribution of the covariates. This also optimizes the bias-variance tradeoff for the given parameter of interest.

Crucially, this makes TMLE a doubly-robust method that will yield unbiased estimates if either the estimated outcome regression or exposure mechanism is consistently estimated [27]. When both the outcome regression and exposure mechanisms are consistently estimated, TMLE is an asymptotically efficient estimator [27]. More technical details on how to obtain targeted maximum likelihood estimates are provided elsewhere [27, 60].

##### Machine learning

To maximize our chances to consistently estimate the outcome regression and the exposure mechanism, we used data-based estimation techniques, a.k.a. ML algorithms [27]. ML algorithms significantly improve the quality of estimation compared to pre-specified parametric models (typically general linear models) which put strong assumptions on data distributions, are likely mis-specified and prone to confirmation biases. Here, we used an ensemble ML algorithm called SuperLearner [63], which we defined as a weighted linear combination of the following basic learners: Linear model with main effects only, Stepwise regression with a step forward procedure, Linear regression with L1-regularization [64], Multivariate adaptive regression splines (MARS) [65], Random Forest (RF) [66].

We used the default hyperparameters of the *SuperLearner* R package for the MARS (inc. maximum degree of interaction = 2) and RF algorithms (inc. number of trees = 500; number of variables to possibly split at in each node = 2; minimum node size = 5 for the outcome regression and 1 for the exposure mechanism). Cross-Validated R^2^ and multi-class Area Under the Receiving Operating Curves were our main indicators of performance. As an example, we report the performances of ML algorithms for the outcome regression and the exposure mechanism when analyzing the association between globalization and the burden of OUD 2019 in Supplementary Table 1 (trimming probabilities of exposure to the 99.9th percentile and not removing outliers).

#### Main analyses

Using the above methodological strategy, we investigated:

1. whether globalization was associated with the burden of OUD 2019;
2. whether globalization was associated with the burden of low back pain and that of other mental and substance use disorders for the same year;
3. whether sub-categories of globalization were associated with the burden of OUD 2019.

#### Sensitivity analyses

Finally, to check the robustness of our results, we ran two other sets of analysis where:

- we used two other levels of truncation for each country’s probability of exposure (truncating probabilities to the 99th and 97.5th percentiles instead of the 99.9th percentiles).
- we removed outlier observations, defined as those with an outcome value inferior to the 1st percentile or superior to the 99th percentile (Supplementary Table 2).

The STROBE for cross-sectional analysis guided the writing of this manuscript [67]. For this country-level analysis that relied on publicly available data, no ethics approval was required. All analyses were performed using R version 4.1 and package *lmtp* [68].

## Results

### Univariate and bivariate analysis

Mean (Standard Deviation) age-standardized 2019 OUD DALYs ranged from 77 (53) in countries with the lowest level of globalization, to 227 (173) in countries with the greatest level of globalization. There was a significant difference in the burden of OUD 2019 across countries with different globalization levels (Table 2). This was also the case for the burden of low back pain, anxiety disorders, cannabis use disorders, and eating disorders 2019 (Table 2). Some of the covariates included in the analysis also varied significantly with globalization, namely the Socio-Demographic Index, Income Inequality, Children Sexual Abuse, Data quality and Healthcare Access & Quality Index (Table 3). Note that, because the standard deviation was relatively high compared to the mean for most variables, we also reported the median and interquartile range, as well as results of non-parametric Kruskal-Wallis tests (Supplementary Tables 3 & 4).

### Multivariate analysis: verification of the positivity assumption

For both our main and sensitivity analyses, the positivity assumption was verified by investigating the mean and maximum values of the inverse probabilities of exposure (a.k.a weights) to various levels of globalization. Across all of our analyses, we found that weights were below 50.1, that is, each country had a probability of exposure of at least 2% (1/50.1). Mean weights were below 4.8, meaning that the mean probability of exposure was at least 21%. Probabilities increased when decreasing the trimming level (i.e. from the 99.9th to the 99th and the 97.5th percentiles), Overall, we concluded that each country had some probability of being exposed to various levels of globalization and that the positivity assumption was not violated in our analyses.

### Association of globalization with the burden of OUD 2019

**Fig. 1:**
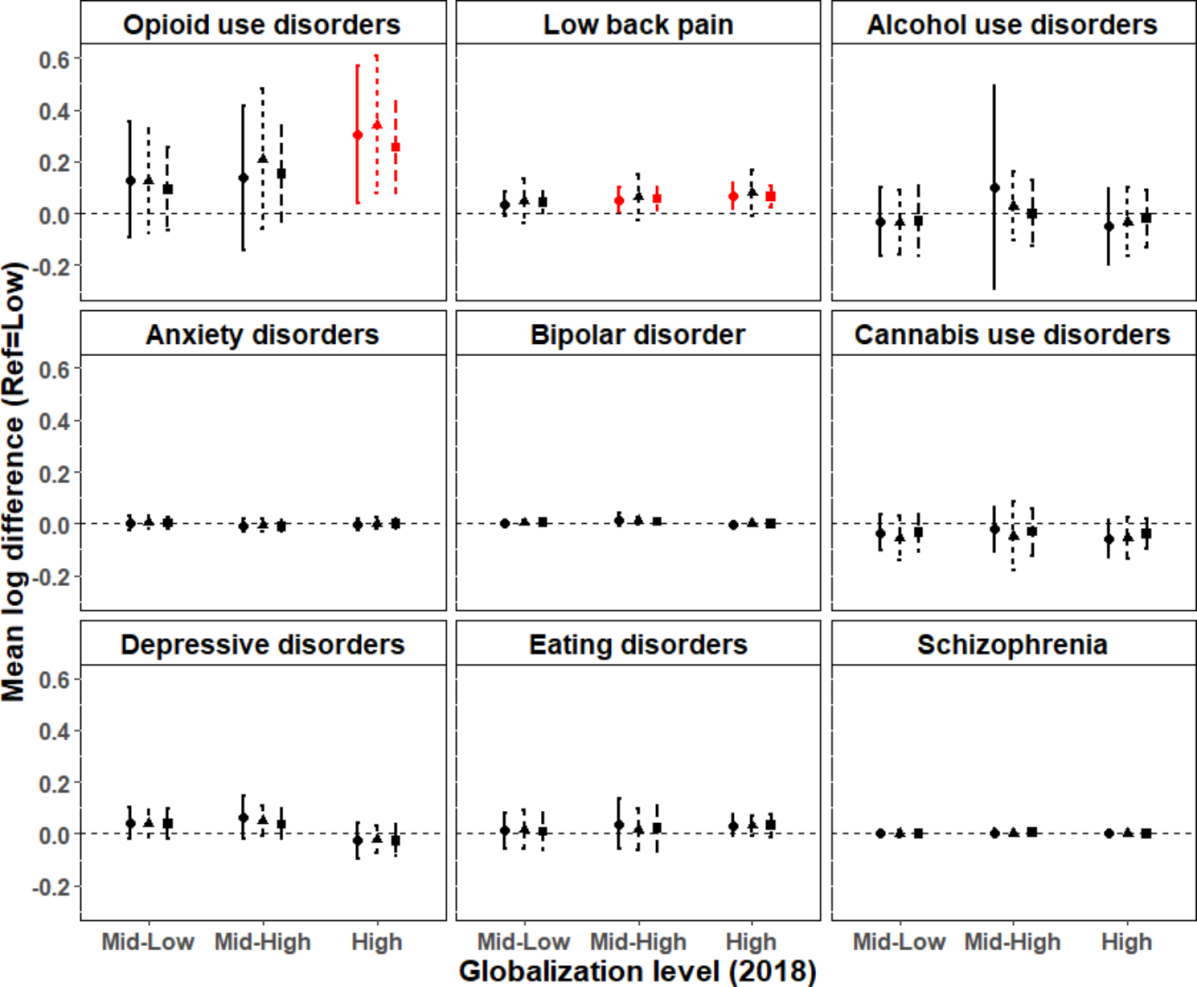
**Association of globalization with the burden of opioid use disorders, low back pain and other mental and substance use disorders 2019.** Mean log differences in 2019 DALYs between each globalization level (Mid-Low, Mid-High and High) vs. the reference (Low). LEGEND. Circles and solid bars: trimming probabilities of exposure to globalization to the 99.9th percentile; triangles and dashed bars: trimming to the 99th percentile; squares and longer dashed bars: trimming to the 97.5th percentile. Error bars denote 95% confidence intervals (CI). Significant differences are shown in red

Taking into account potential country-level confounders, we found that countries with the highest level of globalization were associated with a 31% increase in 2019 OUD DALYs compared to those with the lowest level of globalization (mean log difference High vs. Low: 0.31; 95%CI: 0.04,0.57; p = 0.02; Fig. 1, top-left panel). Countries with other levels of globalization did not show any significant difference compared to those with the lowest level of globalization (Mid-High vs. Low: 0.14; 95%CI: -0.14,0.42; p = 0.33; Mid-Low vs. Low: 0.13; 95%CI: -0.09,0.35; p = 0.26). These results were confirmed when probabilities of exposure were trimmed to different percentiles (Fig. 1, top-left panel), and when outlier countries were removed from the analysis (Supplementary Fig. 1, top-left panel).

### Association of globalization with the burden of low back pain and other MBD

Compared to the reference level (Low), there was a significant effect of the High and Mid-High levels of globalization on 2019 DALYs for low back pain (mean log difference High vs. Low: 0.07; 95%CI: 0.02,0.12; p = 0.007; Mid-High vs. Low: 0.05; 95%CI: 0.00,0.10; p = 0.03; Fig. 1, top-middle panel). However, there were no significant effects of globalization (High vs. Low; Mid-High vs. Low; Mid-Low vs. Low) on 2019 DALYs when the burden of other mental and substance use disorders was used as outcome measures (Fig. 1, top-right panel, center and bottom panels). In general, these results were confirmed when probabilities of exposure were trimmed to different percentiles, and when outlier countries were removed from the analysis. In particular, there was a significant effect of High and Mid-High levels of globalization on 2019 DALYs for low back pain when trimming probabilities of exposure to the 97.5th but not the 99th percentile (Fig. 1, top-middle panel). When outlier countries were removed from the analysis, we found a significant effect of the highest level of globalization only, but at the 99.9th, 99th and 97.5th trimming levels (Supplementary Fig. 1, top-middle panel). Please refer to Fig. 1 and Supplementary Fig. 1 for additional results of our sensitivity analyses.

### Association of sub-indices of globalization with the burden of OUD 2019

**Fig. 2:**
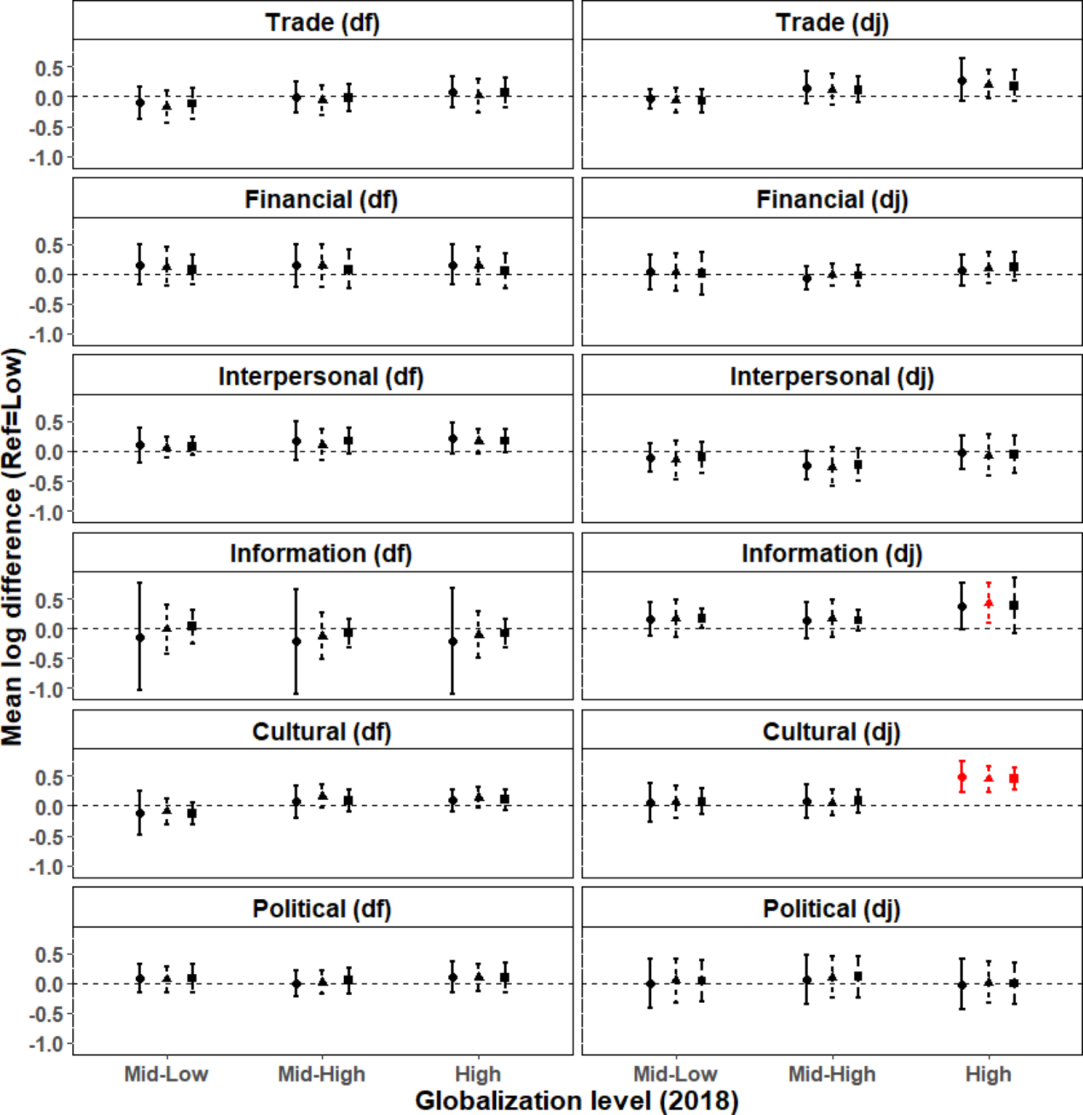
**Association of various sub-indices of globalization with the burden of opioid use disorders 2019.** Mean log differences in 2019 DALYs between each globalization level (Mid-Low, Mid-High and High) vs. the reference (Low). LEGEND. Circles and solid bars: trimming probabilities of exposure to globalization to the 99.9th percentile; triangles and dashed bars: trimming to the 99th percentile; squares and longer dashed bars: trimming to the 97.5th percentile. Error bars denote 95% confidence intervals (CI). Significant differences are shown in red. df: *de facto*; dj: *de jure*

Among sub-segments of economic (trade and financial flows), social (interpersonal contacts, information flows and cultural globalization) and political globalization, only countries with the highest level of socio-cultural *de jure* globalization had significantly greater 2019 OUD DALYs compared to those with the lowest level (mean log difference, High vs. Low: 0.49; 95%CI: 0.23,0.75; p < 0.001; Fig. 2). Other sub-indices did not show any significant effect at any level of globalization. In general, these results were confirmed when trimming probabilities of exposure to the 99th and 97.5th percentiles (Fig. 2), and after removing outlier observations (Supplementary Fig. 2). Please refer to Fig. 2 and Supplementary Fig. 2 for additional results of our sensitivity analyses.

## Discussion

Our results indicate that countries with the highest level of globalization had a greater burden of OUD than countries with the lowest level of globalization. This confirms previous opinions and personal views that the opioid crisis is at risk of dissemination worldwide [3–5, 17]. In particular, these reports have emphasized that the opioid crisis could result from an aggregation of risk factors that we have argued may be specific to globalized countries, including over-prescription of opioids, pressure from the pharmaceutical industry, aging and chronic disease burden (such as pain disorders), and structural socio-economic changes that predispose to feelings of despair.

Unsurprisingly, we found that globalization was also associated with the burden of low back pain. Pain has previously been incriminated as a prominent factor in (1) the US opioid epidemic, explaining in part the rise of opioid prescriptions [69]; and (2) deaths of despair [70, 71]. Pain, and specifically occupational pain, has also been modeled as one of the key mediators between socio-economic changes that have accompanied globalization ideologies and OUD in the US [17]. Therefore, an idea for further research would be to test whether pain is a strong mediator of the relationship between globalization and OUD.

While some of the risk factors of OUD may be shared by other psychiatric and addiction conditions (e.g. despair), we found that OUD was the only mental and substance use disorder related to globalization. The specific link between OUD and globalization needs confirmation, but may reflect the subtle interaction of risk factors that predispose to OUD, where increased opioid prescriptions play a major role [4]. As an alternative, but not mutually exclusive hypothesis, effective psychotropic treatments and psychosocial rehabilitation programs [72], which globalized countries have wide access to, may attenuate the impact of despair on the burden of other mental and substance use disorders. Likewise, other factors not investigated in the current study may have a greater impact than globalization on some mental and behavioral disorders, for instance socio-cultural norms and attitudes towards alcohol in the case of alcohol use disorders [73]. Finally, other mental and substance use disorders may also be impacted by globalization, but in a different way. If increased psychotropic prescriptions are a key characteristic of globalized countries, then mental and substance use disorders that are mostly treated pharmacologically may be associated with other iatrogenic disorders, such as obesity, diabetes, or sexual dysfunctions [74].

A crucial aim of the current study was to determine which facet(s) of globalization (economic, socio-cultural or political) was(were) related to OUD. We retrieved a specific link between OUD and cultural *de jure* globalization, which encompasses measures of gender equality, human capital and civil rights, and aims to represent the ability of citizens to integrate and connect multiple ideas and cultural perspectives [33]. While openness, freedoms and equality are cardinal values of globalized societies, they may also have their own pitfalls and precipitate to OUD. First, increased individual rights and freedoms may have had unexpected negative effects on opioid consumption, via increased demands and unrestrained prescriptions [75, 76]. Second, globalized societies promote ethnic diversity, which can be perceived as a threat by local communities, with potential effects on well-being [77] and therefore feelings of despair. Third, globalized societies strongly value human capital as the return of education on rationality, efficiency and productivity of human beings [33, 78, 79]. Yet, this may have both economical and social negative consequences. In globalized labor markets, the demand for educated workers has increased and the supply of well-paid jobs for individuals without a college degree has decreased [44]. Moreover, an individual’s human capital has become a marker of social status, implying that the college degree has now become “a condition of dignified work and of social esteem” [18, 80]. In this context, those without a college degree may feel humiliated, abandoned or left aside, and may also express feelings of despair [25, 80]. Overall, while these hypotheses lie on scientific evidence, they are speculative regarding our study design, have mostly been raised or tested in the US, and hence need further confirmation. Carrying out these interpretations with caution, we also wish to clarify that our findings by no means infer that globalized countries should revert to patriarchy, social inequalities or discrimination, nor that these would automatically solve the opioid crisis.

### Limitations

First, our sample included countries and not individuals, hence we have no information as to which societal strata may specifically be impacted by globalization. In the US for instance, several studies have suggested that different populations may be impacted differently by the current opioid epidemic [81–86], which our research cannot confirm. Second, with our data we have no indication as to how globalization (and in particular cultural *de jure* globalization) may be specifically linked to OUD, e.g. via increased prescriptions, higher demands, or heightened feelings of despair. Besides, other aspects of economic and social globalization showed significant effects on OUD in our sensitivity analyses. Whether occupational, economic, and/or social factors such as the level and strength of unionization, occupational industry and disease, social movements of various kinds (or defeated social movements) are key mediators of the relationship between globalization and OUD should be investigated in future studies. Third, we chose to only extract high-quality data as per the GBD study 2019. Further studies should test whether our results are generalizable to other countries not included in this analysis because of lower data-quality standards. Fourth, we cannot ascertain the degree of accuracy of GBD estimates used in our analysis. Accuracy of estimation is highly reliant on data availability and quality of data collection, hence may not be completely flawless, even in countries with high-quality ratings. Therefore, we encourage future research to confirm or infirm our results using other data sources. Fifth, we acknowledge that choosing a one-year lag between our exposure and outcome was an arbitrary choice. Clearly, as little is known on how social determinants interact and accumulate over time to affect health outcomes, this choice was dictated by simplicity and convenience with respect to our analytical strategy.

### Conclusion

Previous accounts relating globalization to OUD mostly relied on within-countries observations or opinions and personal views. Here, we demonstrate for the first time that this relationship does indeed exist in a quantitative country-based analysis. Moreover, we show that this relationship is specifically related to cultural aspects of globalization, which summarize measures of gender equity, human capital and civil rights. Should this finding be replicated, it would not only plead for better regulation of opioid prescriptions, but also call into question the erosion of local cultures and the over-reliance of globalized societies on high achievement in education.

Finally, it is important to note that a number of countries, mostly non-globalized low or middle-income countries, are characterized by undertreatment of pain and underutilization of opioids. We wish to state that our results should not contribute to the so-called “opiophobia”, the fear of prescribing opioids due to its potential adverse effects [87, 88]. Better education of health professionals, patients and their families should enforce the message that opioids are safe drugs when prescribed properly.

**Table 1:** Countries included in the analysis, sorted according to their globalization level

Globalization level			
Low [41, 64]^a^ N = 23	Mid-Low (64,72]^a^ N = 23	Mid-High (72,82.5]^a^ N = 19	High (82.5,91]^a^ N = 22
-Bahamas -Belize -Brazil -Brunei Darussalam -China -Colombia -Cuba -Ecuador -Guatemala -Guyana -Jamaica -Kazakhstan -Kyrgyzstan -Nicaragua -Paraguay -Sri Lanka -Suriname -Syrian Arab Republic -Tajikistan -Trinidad and Tobago -Turkmenistan -Uzbekistan -Venezuela (Bolivarian Republic of)	-Albania -Argentina -Armenia -Azerbaijan -Bahrain -Belarus -Costa Rica -Dominican Republic -El Salvador -Georgia -Iceland -Kuwait -Mauritius -Mexico -North Macedonia -Panama -Peru -Philippines -Republic of Moldova -Russian Federation -South Africa -Thailand -Turkey	-Australia -Bulgaria -Chile -Croatia -Israel -Japan -Latvia -Lithuania -Malta -New Zealand -Poland -Republic of Korea -Romania -Serbia -Slovakia -Slovenia -Ukraine -United States of America -Uruguay	-Austria -Belgium -Canada -Czechia -Denmark -Estonia -Finland -France -Germany -Greece -Hungary -Ireland -Italy -Luxembourg -Netherlands -Norway -Portugal -Singapore -Spain -Sweden -Switzerland -United Kingdom

^a^Lower and upper bounds of Globalization Index as per the KOF Swiss Economic Institute [33]

**Table 2:** Burden of disease 2019 and 1990^a^ for the disorders included in the study

Disorder	Year	Globalization level				P value^b^
		Low	Mid-Low	Mid-High	High	
Opioid Use Disorders	2019	77 (53)	103 (74)	210 (326)	227 (173)	0.01
	1990	68 (35)	93 (94)	97 (84)	122 (82)	0.15
Low Back Pain	2019	778 (82)	858 (138)	1067 (137)	1037 (124)	< 0.001
	1990	799 (66)	876 (162)	1113 (177)	1090 (158)	< 0.001
Alcohol Use Disorders	2019	312 (195)	307 (270)	350 (185)	320 (128)	0.91
	1990	358 (310)	363 (335)	386 (219)	326 (134)	0.91
Anxiety Disorders	2019	383 (129)	382 (77)	400 (126)	502 (135)	0.002
	1990	369 (110)	372 (77)	396 (118)	495 (126)	< 0.001
Bipolar Disorder	2019	161 (52)	145 (47)	156 (62)	178 (31)	0.15
	1990	161 (52)	144 (46)	154 (60)	178 (31)	0.13
Cannabis Use Disorders	2019	10 (5.3)	7.1 (2.4)	13 (6.6)	15 (7.4)	< 0.001
	1990	9.7 (5.2)	7.0 (2.3)	14 (8.4)	17 (7.3)	< 0.001
Depressive Disorders	2019	584 (149)	555 (128)	556 (148)	633 (133)	0.22
	1990	606 (146)	574 (138)	587 (165)	697 (132)	0.03
Eating Disorders	2019	42 (16)	45 (20)	67 (46)	93 (25)	< 0.001
	1990	37 (17)	37 (16)	54 (35)	78 (22)	< 0.001
Schizophrenia	2019	178 (10)	180 (12)	199 (26)	182 (20)	< 0.001
	1990	176 (8.9)	177 (11)	197 (28)	181 (22)	0.001

^a^Mean (SD) age-standardized Disability Adjusted Life Years rates (per 100,000 inhabitants). ^b^One-way between-group ANOVA

**Table 3:** Description of the covariates

Variables Mean (SD)	Globalization level				P value^f^
	Low	Mid-Low	Mid-High	High	
Socio-Demographic Index^a^	0.65 (0.077)	0.70 (0.072)	0.80 (0.048)	0.85 (0.048)	< 0.001
Unemployment rate^b^	6.5 (3.1)	7.7 (6.8)	5.9 (2.5)	6.5 (4.0)	0.62
Income Inequality^c^	0.46 (0.051)	0.44 (0.098)	0.39 (0.075)	0.34 (0.037)	< 0.001
Urbanization Index	0.36 (0.11)	0.39 (0.15)	0.43 (0.20)	0.44 (0.17)	0.31
Children Sexual Abuse^d^	4.3 (2.0)	6.1 (2.9)	7.3 (2.2)	6.6 (1.3)	< 0.001
Data quality^e^	3.74 (0.69)	3.74 (0.81)	4.26 (0.73)	4.59 (0.50)	< 0.001
Healthcare Access & Quality Index	61 (8.2)	68 (11)	83 (8.1)	93 (4.0)	< 0.001

^a^Composite indicator of income per capita, access to education and fertility. ^b^Percentage of the labor force. ^c^Ratio of individuals whose income belongs to the top 10% of the population, divided by the entire population. ^d^Age-standardized summary of exposure value (SEV, %). ^e^Using the 5 stars quality rating system from the GBD database. ^f^One-way between-group ANOVA

### Electronic supplementary material

Below is the link to the electronic supplementary material.

Supplementary Material 1

## Data Availability

GB had full access to all the data in the study and takes responsibility for the integrity of the data and the accuracy of the data analysis. The datasets generated during and/or analyzed during the current study are available upon request to the corresponding author, GB.
