# Supplementary material for: Association of globalization with the burden of opioid use disorders 2019. A country-level analysis using targeted maximum likelihood estimation
Source: Global Health. 2023 Oct 16;19:76. doi: 10.1186/s12992-023-00980-3 (PMC10577998; doi:10.1186/s12992-023-00980-3)
Supplement: Supplementary file 1 — Supplementary Material 1 [file 12992_2023_980_MOESM1_ESM.docx]

Supplementary Materials

Barbalat G, Reddy G, Franck N. Association of globalization with the burden of opioid use disorders 2019. A country-level analysis using targeted maximum likelihood estimation.

Supplementary Table 1. Performance of machine learning algorithms for the exposure mechanism and the outcome regression: example.

Supplementary Table 2. Outlier observations for the outcome variables.

Supplementary Table 3. Non-parametric tests on burden of disease 2019 and 1990 for the disorders included in the study.

Supplementary Table 4. Non-parametric tests on the covariates.

Supplementary Figure 1. Globalization and burden of mental and substance use disorders 2019 after removing outlier observations.

Supplementary Figure 2. Sub-indices of globalization and burden of opioid use disorders 2019 after removing outlier observations.

Supplementary Table 1. Performance of machine learning algorithms for the exposure mechanism and the outcome regression: example^a^.

| Algorithm | Outcome Regression^b^ | | Exposure Mechanism^c^ | |
| --- | --- | --- | --- | --- |
|  | Coefficient | Performance (mean CV-R^2^) | Coefficient | Performance (mean CV-AUC) |
| Linear Model | 0.31 | 0.68 | 0.01 | 0.73 |
| Stepwise | 0.04 | 0.65 | 0.23 | 0.75 |
| LASSO | 0.05 | 0.67 | 0.03 | 0.74 |
| MARS | 0.14 | 0.64 | 0.21 | 0.76 |
| Random Forest | 0.46 | 0.69 | 0.52 | 0.84 |
| SuperLearner^d^ | - | 0.71 | - | 0.82 |

^a^In the analysis where globalization is the exposure and the burden of OUD 2019 is the outcome, trimming probabilities of exposure to the 99.9^th^ percentile and not removing outliers

^b^Fits the covariates on 2019 OUD DALYs across the four levels of globalization with a SuperLearner ensemble (defined below). Performance is measured by the Cross-Validated R^2^ (20 folds for cross-fitting)

^c^Fits the covariates on the exposure across the four levels of globalization with a SuperLearner ensemble (defined below). Performance is measured by the multiclass Cross-Validated Area Under the Receiving Operating Curve (20 folds for cross-fitting)

^d^Defined as a weighted linear combination (that would sum up to 1) of the following basic learners: Linear model with main effects only (Linear Model), Stepwise regression with a step forward procedure (Stepwise), Linear regression with L1-regularization (LASSO), Multivariate adaptive regression splines (MARS), Random Forest. For each algorithm, we used the default hyperparameters of the *SuperLearner* R package

LEGEND. CV: Cross-Validated; AUC: Area Under the receiving operating Curve. Cross-Validated R^2^ and AUC are summary measures of model performance (range: 0-1; values closer to 1 indicate better performance).

Supplementary Table 2. Outlier observations^a^ for the outcome variables.

| Disorder | Outliers | |
| --- | --- | --- |
|  | < 1^st^ pct | > 99^th^ pct |
| Opioid use disorders | Singapore  (33.8) | United States of America (1481.4) |
| Low back pain | China  (579.1) | United States of America (1402.9) |
| Alcohol use disorders | Kuwait  (46.6) | Belarus  (1007.6) |
| Anxiety disorders | Uzbekistan  (194.1) | Portugal  (767.7) |
| Bipolar disorder | China  (39.6) | New Zealand  (324.1) |
| Cannabis use disorders | Turkey  (2.5) | Canada  (35.1) |
| Depressive disorders | Brunei Darussalam  (260.3) | Greece  (927.8) |
| Eating disorders | Tajikistan  (19.2) | Australia  (218.7) |
| Schizophrenia | South Africa  (141.1) | United States of America  (270.7) |

^a^ Expressed as age-standardized Disability Adjusted Life Years rates (per 100000 inhabitants)

Supplementary Table 3. Non-parametric tests on burden of disease 2019 and 1990^a^ for the disorders included in the study.

| Disorder | Year | Globalization level | | | | P value^b^ |
| --- | --- | --- | --- | --- | --- | --- |
|  |  | Low | Mid-Low | Mid-High | High |  |
| Opioid Use Disorders | 2019 | 63 (30) | 79 (51) | 97 (171) | 195 (179) | <0.001 |
|  | 1990 | 61 (33) | 62 (42) | 64 (76) | 115 (89) | 0.12 |
| Low Back Pain | 2019 | 800 (92) | 809 (211) | 1086 (140) | 1061 (188) | <0.001 |
|  | 1990 | 795 (99) | 819 (253) | 1115 (118) | 1080 (184) | <0.001 |
| Alcohol Use Disorders | 2019 | 254 (254) | 243 (123) | 323 (144) | 332 (101) | 0.22 |
|  | 1990 | 288 (214) | 263 (194) | 368 (212) | 323 (152) | 0.60 |
| Anxiety Disorders | 2019 | 394 (127) | 368 (100) | 343 (191) | 494 (183) | 0.01 |
|  | 1990 | 386 (104) | 362 (93) | 336 (173) | 494 (177) | 0.004 |
| Bipolar Disorder | 2019 | 180 (85) | 118 (74) | 119 (90) | 191 (32) | 0.17 |
|  | 1990 | 180 (85) | 119 (74) | 118 (85) | 190 (32) | 0.14 |
| Cannabis Use Disorders | 2019 | 8.1 (8.7) | 6.6 (3.5) | 12 (4.4) | 14 (9.4) | <0.001 |
|  | 1990 | 8.1 (8.3) | 6.5 (3.1) | 11 (5.8) | 15 (10) | <0.001 |
| Depressive Disorders | 2019 | 578 (154) | 541 (211) | 520 (248) | 620 (169) | 0.22 |
|  | 1990 | 568 (159) | 542 (225) | 591 (289) | 692 (223) | 0.03 |
| Eating Disorders | 2019 | 41 (15) | 35 (32) | 40 (48) | 97 (15) | <0.001 |
|  | 1990 | 35 (15) | 33 (20) | 35 (35) | 86 (20) | <0.001 |
| Schizophrenia | 2019 | 177 (8.0) | 181 (8.3) | 190 (13) | 178 (15) | <0.001 |
|  | 1990 | 175 (6.0) | 179 (6.9) | 187 (16) | 178 (14) | <0.001 |

^a^Median (IQR) age-standardized Disability Adjusted Life Years rates (per 100000 inhabitants)

^b^Kruskal-Wallis test (non-parametric alternative to the one-way ANOVA test)

Supplementary Table 4. Non-parametric tests on the covariates.

| Variables  Median  (IQR) | Globalization level | | | | P value^f^ |
| --- | --- | --- | --- | --- | --- |
|  | Low | Mid-Low | Mid-High | High |  |
| Socio-Demographic Index^a^ | 0.63 (0.069) | 0.69 (0.066) | 0.80 (0.077) | 0.85 (0.052) | <0.001 |
| Unemployment rate^b^ | 6.5 (4.2) | 4.9 (7.0) | 5.2 (3.4) | 5.5 (3.0) | 0.9 |
| Income Inequality^c^ | 0.49 (0.076) | 0.46 (0.16) | 0.37 (0.097) | 0.33 (0.034) | <0.001 |
| Urbanization Index | 0.37 (0.15) | 0.38 (0.22) | 0.38 (0.31) | 0.42 (0.16) | 0.433 |
| Children Sexual Abuse^d^ | 4.2 (2.3) | 5.6 (2.8) | 6.7 (1.4) | 6.7 (1.1) | <0.001 |
| Data quality^e^ | 1.0 (1.0) | 1.0 (1.0) | 1.0 (1.0) | 2.0 (1.0) | <0.001 |
| Healthcare Access & Quality Index | 59 (7.7) | 68 (12) | 83 (14) | 94 (4.1) | <0.001 |

^a^Composite indicator of income per capita, access to education and fertility

^b^Percentage of the labor force

^c^Ratio of individuals whose income belongs to the top 10% of the population, divided by the entire population

^d^Age-standardized summary of exposure value (SEV, %)

^e^Using the 5 stars quality rating system from the GBD database

^f^Kruskal-Wallis test (non-parametric alternative to the one-way ANOVA test)

Supplementary Figure 1. Globalization and burden of mental and substance use disorders 2019 after removing outlier observations.


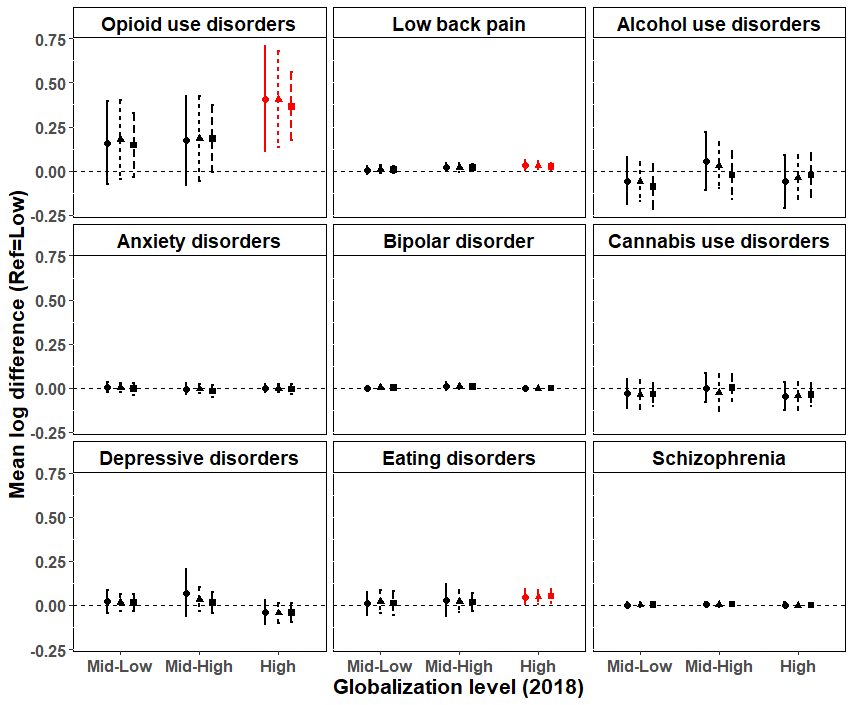


Mean log differences in 2019 DALYs between each globalization level (Mid-Low, Mid-High and High) vs. the reference (Low).

LEGEND. Circles and solid bars: trimming probabilities of exposure to globalization to the 99.9^th^ percentile; triangles and dashed bars: trimming to the 99^th^ percentile; squares and longer dashed bars: trimming to the 97.5^th^ percentile. Error bars denote 95% confidence intervals (CI). Significant differences are shown in red.

df: *de facto*; dj: *de jure*

Supplementary Figure 2. Sub-indices of globalization and burden of opioid use disorders 2019 after removing outlier observations.


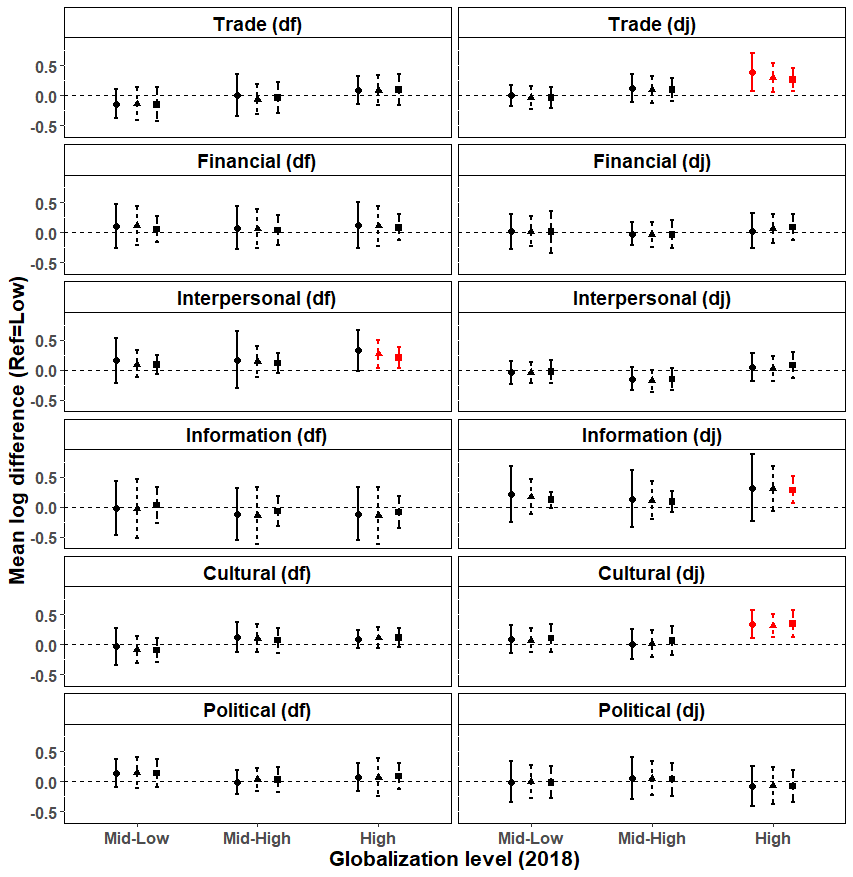


Mean log differences in 2019 DALYs between each globalization level (Mid-Low, Mid-High and High) vs. the reference (Low).

LEGEND. Circles and solid bars: trimming probabilities of exposure to globalization to the 99.9^th^ percentile; triangles and dashed bars: trimming to the 99^th^ percentile; squares and longer dashed bars: trimming to the 97.5^th^ percentile. Error bars denote 95% confidence intervals (CI). Significant differences are shown in red.

df: *de facto*; dj: *de jure*
